# Cerebral-cognitive reserve: concept and functions of the cerebral-cognitive reserve

**DOI:** 10.1192/j.eurpsy.2024.1417

**Published:** 2024-08-27

**Authors:** A. Sidenkova

**Affiliations:** Psychiatry, Ural State Medical University, Yekaterinburg, Russian Federation

## Abstract

**Introduction:**

The modern understanding of AD allows us to consider it through the constructs of “vulnerability” and “stability” of the brain as a dynamic system of dialectical interaction between the pathogenic process and the protective process that prevents neurodegeneration. The concept of cognitive reserve (CR) is based on observations of discrepancy between the degree of brain pathology and the severity of clinical manifestations. The concept of “reserve” was proposed to describe the resistance of the brain to a developing damage caused by a pathological process. Stern (2002) considered CR as a protective factor that modifies the impact of brain pathology on cognitive function. The researchers have defined CR as an ability to optimize cognitive function through differential involvement of structures or neural networks of the brain into brain activity.

**Objectives:**

A systematic review of scientific studies has been conducted

**Methods:**

The review includes an analysis of full-text literature sources.

**Results:**

Several possible directions of CR influence on cognitive functions have been described:

1. CR may reduce the risk of MCI or dementia through mechanisms, which do not depend on the level of neurodegenerative pathological changes in the brain.
2. CR can interact with the markers of brain pathology or healthaffectingthe future cognitive decline or risk of progression. It has been found that smaller volumes or thickness in some AD vulnerable areas of the brain represent a stronger risk factor for cognitive impairment in people with low CR than in people with higher CR. CR protective effects on clinical outcomes reduce as the number of damaged neurons increases.
3. The protective effect of CR increasesduring late AD onset and at a low rate of the damaged substrate accumulation.
4. CR changes the relationship between genetic factors and aging withclinical and cognitive outcomes. The relationship between age and AD pathology level or age-related structural changes in the brain may weaken in people with higher CR

**Conclusions:**

The concept of cerebral-cognitive reserve actualizes the problem of the search for compensatory mechanisms of cognitive deficit in AD, the assessmentof the structure of the reserve, the development and implementationof programs to maintain the reserve, the preventionof its depletion, starting from the preclinical stage of the disease, which can prevent the transformation of preclinical manifestations of AD into cognitive disorders

**Disclosure of Interest:**

None Declared

